# Neuroprotective effects of low-dose ionizing radiation in diabetic rats exposed to chronic unpredictable stress

**DOI:** 10.5455/javar.2026.m1033

**Published:** 2026-03-24

**Authors:** Nayanatara Arun Kumar, Megha Gokul, Harini Narayanam, Shyamala Nayak, Avin Kumar, Kamalaksh Shenoy

**Affiliations:** 1 Department of Physiology, Kasturba Medical College Mangalore, Manipal Academy of Higher Education, Manipal, India; 2 Department of Physiology, Faculty of Medicine, Manipal University College, Melaka, Malaysia; 3 Department of Biochemistry, SSPM Medical College and Lifetime Hospital, Kasal, Sindhudurg 416534, Maharashtra, India; 4 Department of Radiation Oncology, A J Hospital and Research Centre, Mangalore, Karnataka, India

**Keywords:** Diabetes mellitus, chronic stress, neuroprotection, ionizing radiation, neurotransmitters, hippocampus, corticosterone, depression, experimental animal models

## Abstract

**Objectives:** Diabetes mellitus combined with prolonged stress has been linked with neurochemical and functional alterations that may alter brain function. The present study was designed to test the hypothesis that low-dose ionizing radiation (LDIR) may exert neuroprotective effects in diabetic rats exposed to chronic unpredictable stress (CUS) by improving neurochemical and neuroendocrine alterations.

**Materials and Methods:** Diabetes was induced in Wistar rats using streptozotocin (40 mg/kg, intraperitoneally), followed by exposure to CUS and LDIR. Radiation dose (0.1 Gy per session, three times weekly for 15 days) was administered. Behavioral assessments were conducted using open field, rotarod, and object recognition tests. Serum corticosterone levels were measured to evaluate stress-associated neuroendocrine changes. Neurotransmitter levels, including dopamine, serotonin, noradrenaline, and acetylcholine, were quantified in the hippocampus, cerebral cortex, and cerebellum using ELISA.

**Results:** Diabetic rats exposed to CUS showed a significant increase in serum corticosterone levels, reduced neurotransmitter concentrations, and impaired behavioral performance compared with control animals. Treatment with LDIR was associated with significant improvements in corticosterone levels, neurotransmitter concentrations, and behavioral performance compared with untreated diabetic-stressed animals. Combined treatment with LDIR and paroxetine demonstrated additional improvements in selected parameters.

**Conclusions:** These findings demonstrate that LDIR exerts neuroprotective effects in diabetic rats exposed to CUS and suggest its potential as a non-invasive, adjunctive therapeutic approach to mitigate stress-related neuropsychiatric complications associated with diabetes.

## 1. Introduction

Diabetes mellitus and its associated neurological complications have emerged as major global health concerns. Depression in individuals with diabetes is not merely a psychological comorbidity but a clinically significant condition that can impair glycemic control, increase the risk of complications, and contribute to elevated mortality rates [1, 2]. Despite its clinical relevance, depression remains one of the most neglected manifestations of diabetes. Diabetes and depressive disorders influence each other through a complex interplay of biological, behavioral, and psychosocial mechanisms. Chronic inflammation, oxidative stress, and adverse lifestyle factors are considered key contributors to this comorbidity [3]. Furthermore, the psychological and physiological burden of diabetes-related stress can intensify depressive manifestations, establishing a self-perpetuating cycle that adversely affects both metabolic regulation and mental health. Therefore, integrated therapeutic approaches that address both metabolic dysfunction and stress-related neurobiological alterations are essential to improve disease outcomes and reduce the overall burden of illness [4, 5].

Experimental animal models are essential for understanding the pathophysiological mechanisms underlying depression in diabetes. The CUS model is widely employed to mimic the duration, variability, and unpredictability of stress exposure encountered in human life. This model reliably induces depressive-like behaviors in rodents and reproduces neurochemical and behavioral alterations relevant to depression [6].

Reports from recent research works emphasize the biological role of low-dose ionizing radiation, which differs markedly from the harmful effects observed at higher doses. Increasing evidence suggests that low-dose radiation may induce adaptive responses with potential therapeutic benefits in various disease conditions [7]. LDIR has been shown to modulate oxidative stress, enhance mitochondrial function, and stimulate endogenous antioxidant defense mechanisms, thereby reducing neuroinflammatory responses [8]. However, the neuroprotective effects of LDIR in complex metabolic and neuropsychiatric disorders such as diabetic depression remain poorly understood. We hypothesized that low-dose ionizing radiation would exert neuroprotective effects in diabetic rats exposed to chronic unpredictable stress by improving neurotransmitter homeostasis and mitigating stress-associated neurobehavioral and neuroendocrine alterations. Understanding the interplay between diabetes, chronic stress, and neuroprotective strategies may facilitate the development of novel therapeutic interventions.

Therefore, this study was commenced to explore the neuroprotective effects of LDIR in a diabetic stress-induced Wistar rat model. Neurobehavioral, cognitive, and sensorimotor functions were evaluated, along with alterations in key neurotransmitters targeting the hippocampus, cerebral cortex, and cerebellum. Collectively, the findings underscore the therapeutic relevance of LDIR as an adjunct modality capable of enhancing neurological outcomes in diabetes-induced depressive conditions.

## 2. Materials and Methods

Adult Wistar rats of either sex (4–5 months old; 150–220 gm) were procured from the institutional animal house under standard laboratory conditions (12 h light–dark cycle). Animals had free access to a standard pellet diet and water, except during specific stress procedures.

### 2.1. Ethical approval

The study was conducted after obtaining approval from the institutional animal ethics committee. All the procedures were followed in accordance with the ethical standards for animal research (KMC/MNG/IAEC/24-2024).

### 2.2. Sex distribution and randomization

Both male and female Wistar rats were included in this study to boost the translational relevance and generalizability of the findings. Animals were randomly assigned to experimental groups, with equal representation of males and females (*n* = 3 males and 3 females per group). Estrous cycle monitoring was not performed, as the primary objective was to assess the overall neuroprotective effects of low-dose ionizing radiation under diabetic stress conditions rather than sex-specific hormonal influences.

### 2.3. Induction of diabetes

Diabetes was induced by a single intraperitoneal injection of streptozotocin at a dose of 40 mg/kg body weight. Streptozotocin was freshly prepared in a cold 10 mM sodium citrate buffer (pH 4.5) immediately prior to administration to maintain stability. Animals were fasted overnight (12 h) before injection to enhance the efficacy of diabetes induction. Fasting blood glucose levels were measured 72 h after STZ administration using a validated glucometer. Rats with fasting blood glucose levels greater than 200 mg/dl were considered diabetic and included in the study [9]. The rats belonging to the control group received an equivalent volume of citrate buffer alone. The fasting period was limited to 12 h and was applied uniformly to all animals to minimize variability and reduce its potential impact as a confounding stress factor.

### 2.4. CUS protocol

The stress protocol consisted of chronic unpredictable stressors of varying intensity and duration; therefore, the term “Chronic Unpredictable Stress (CUS)” was used rather than “Chronic Unpredictable Mild Stress (CUMS).” CUS was induced according to established protocols, with minor modifications [10, 11]. Experimental animals were exposed to a series of variable and unpredictable stressors for 15 consecutive days. Stressors were administered at different times each day to minimize habituation and enhance unpredictability. Behavioral testing was initiated 24 h after completion of the CUS protocol to avoid the influence of acute stress. The stress paradigm included restraint stress in ventilated plastic restrainers (1 h), rotation stress using a rotating spinner (50 rpm, 1 h), wet bedding with 5 cm of wet husk (4 h), warm and cold swim stress in a cylindrical tank (45°C for 30 min or 8°C for 20 min), and intermittent foot shocks (1.5 mA for 10 min) delivered through a grid floor chamber. Additional stressors included tail pinch (20 min), isolation housing (4 h), overcrowding, cage tilting at 45°, reversal of the light–dark cycle, exposure to hot or cold air, and overnight food or water deprivation according to the stress schedule.

### 2.5. LDIR exposure

**Figure 1.**
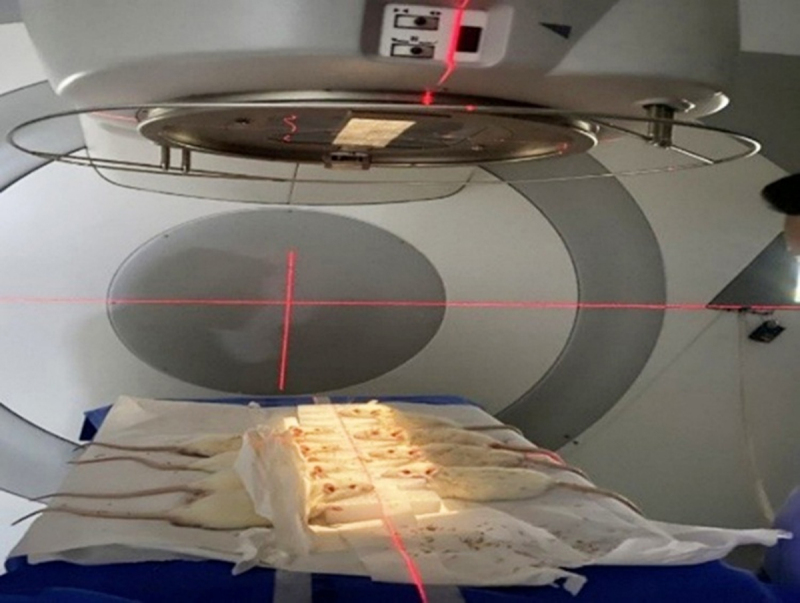
Wistar rats exposed to LDIR.

Low-dose ionizing radiation exposure was performed using a standardized experimental setup and delivered using a medical linear accelerator at the Department of Radiation Oncology, AJ Institute of Medical Sciences and Research Centre, Mangalore. Each rat received a radiation dose of 0.1 Gy per session, delivered three times per week over 15 days, for a total cumulative dose of 0.6 Gy (Figure 1). This dose was selected based on previous experimental evidence demonstrating that cumulative radiation doses below 1 Gy induce adaptive cellular and neuroprotective responses without causing structural or functional damage to neural tissue.

Animals were anesthetized using ketamine (80 mg/kg, intraperitoneal) and xylazine (10 mg/kg, intraperitoneal) prior to irradiation to ensure immobilization and minimize procedural stress. To eliminate anesthesia as a confounding variable, animals in other experimental groups were also subjected to the same anesthesia protocol without radiation exposure. Rats were positioned on the treatment couch, and radiation was delivered specifically to the cranial region using anteroposterior and posteroanterior beams, each contributing 50% of the total dose, to ensure targeted exposure to brain tissue while minimizing systemic radiation exposure. The selected dose and schedule were designed to allow sufficient recovery between exposures and to promote adaptive neuroprotective responses via radiation hormesis. All irradiation procedures were performed under standardized conditions to ensure consistency across experimental animals.

### 2.6. Antidepressant treatment

Paroxetine (10 mg/kg/day) was given following each day of CUS exposure, as described previously [12].

### 2.7. Experimental design and animal grouping

Rats were randomly assigned to groups according to the treatment protocol. Each group had six animals (*n* = 6).

Group I (Control): The rats in this group were not exposed to any stress or treatment procedures.

Group II (Diabetes): This group of rats received a single injection of streptozotocin.

Group III (Diabetic stress): This group of diabetic rats underwent 15 days of CUS.

Group IV (Diabetic stress + LDIR treated): This group of rats was treated with LDIR for 3 days/week for 15 days.

Group V: In this group, the included diabetic rats exposed to CUS were treated with antidepressants and exposed to LDIR.

### 2.8. Behavioral, cognitive, and motor assessments

Behavioral assessments were performed 24 h after completion of the CUS protocol to avoid acute stress effects. Animals were acclimatized to the testing room for 30 min prior to testing. The open field test was conducted in a square arena under controlled lighting conditions to assess exploratory and anxiety-like behavior. The rotarod test was performed using a motorized rotating rod apparatus to evaluate motor coordination and balance. Cognitive function was assessed using the object recognition test in a controlled environment. All behavioral tests were conducted during the light phase under standardized conditions [13, 14].

### 2.9. Neurochemical analysis

Immediately after completion of behavioral assessments, blood samples were collected by cardiac puncture under anesthesia for estimation of serum corticosterone levels. Animals were then euthanized, and the brain was rapidly dissected on an ice-cold surface. The hippocampus, cerebral cortex, and cerebellum were isolated and homogenized in ice-cold phosphate buffer (pH 7.4). Neurotransmitter and hormone levels were quantified using commercially available rat-specific ELISA kits according to the manufacturer’s instructions [15, 16]. Serum corticosterone levels were measured using a Rat Corticosterone ELISA Kit (96T, Catalog No: OPK7957). Dopamine levels were measured using the Rat Dopamine ELISA Kit (96T, Catalog No: OPK8953), serotonin using the Rat 5-Hydroxytryptamine (5-HT) ELISA Kit (96T, Catalog No: OPK8954), noradrenaline using the Rat Noradrenaline ELISA Kit (96T, Catalog No: OPK8956), and acetylcholine using the Rat Acetylcholine ELISA Kit (96T, Catalog No: OPK9644). All assays were performed according to manufacturer protocols under standardized laboratory conditions to ensure accuracy and reproducibility.All samples were analyzed in duplicate, and assay procedures were performed under identical experimental conditions to minimize inter-assay variability.

### 2.10. Statistical analysis

Data are expressed as mean ± standard deviation (SD). Statistical analysis was performed using JAMOVI. Normality of data distribution was assessed using the Shapiro–Wilk test. Comparisons among experimental groups were performed using one-way analysis of variance (ANOVA), followed by Tukey’s post hoc multiple comparison test. A value of *p* < 0.05 was considered statistically significant.

## 3. Results

### 3.1. Serum corticosterone levels

**Figure 2.**
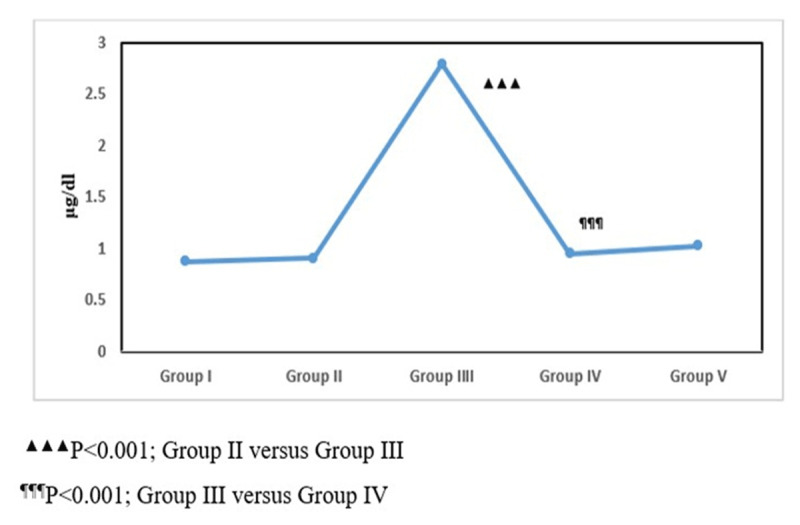
Serum corticosterone levels in experimental groups.

Assessment of endocrine responses revealed significant differences in serum corticosterone concentrations among experimental groups (one-way ANOVA, F (4,25) = 28.15, *p* < 0.001). Control animals (Group I) and diabetic rats without stress exposure (Group II) showed relatively stable corticosterone levels. In contrast, diabetic rats subjected to CUS (Group III) displayed a pronounced elevation in circulating corticosterone levels, reflecting stress-induced activation of the hypothalamic–pituitary–adrenal axis. Exposure to low-dose ionizing radiation (Group IV) markedly attenuated this stress-associated endocrine imbalance, as evidenced by reduced corticosterone levels compared with stressed diabetic animals. Combined treatment with LDIR and antidepressant therapy (Group V) showed further improvement. These findings indicate that LDIR mitigates stress-associated endocrine dysregulation under diabetic conditions (Figure 2).

### 3.2. Blood glucose levels

**Figure 3.**
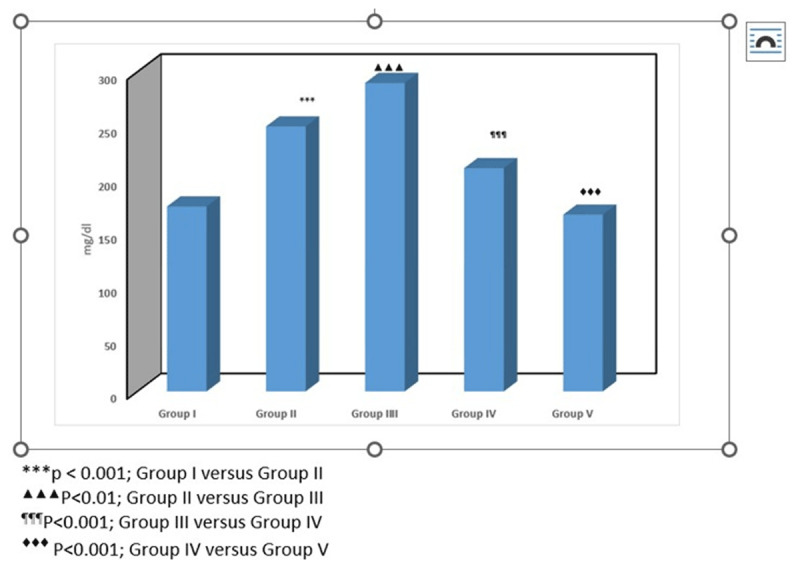
Blood glucose level in the experimental groups.

Fasting blood glucose concentrations differed significantly among experimental groups (one-way ANOVA, F (4,25) = 73.18, *p* < 0.001). Streptozotocin administration resulted in marked hyperglycemia in diabetic animals (Group II), confirming successful induction of diabetes. CUS (Group III) further exacerbated hyperglycemia, indicating an additive metabolic burden. Treatment with low-dose ionizing radiation (Group IV) significantly improved glycemic status compared with stressed diabetic animals. Furthermore, animals receiving combined LDIR and antidepressant therapy (Group V) demonstrated additional improvement in glucose regulation, suggesting enhanced metabolic recovery (Figure 3).

### 3.3. Open field test

**Figure 4.**
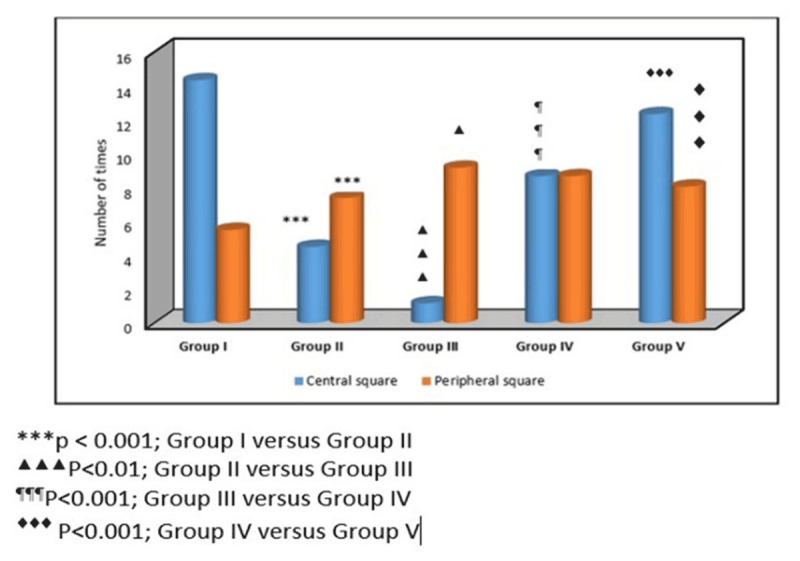
Open field test in the experimental groups.

Exploratory and anxiety-related behaviors differed significantly among experimental groups. The number of central square entries showed significant differences (one-way ANOVA, F (4,25) = 40.13, *p* < 0.001). Diabetic rats exhibited reduced central square exploration compared with control animals, indicating increased anxiety-like behavior. CUS further exacerbated this reduction. Treatment with low-dose ionizing radiation significantly improved exploratory behavior, as evidenced by increased entries into the central square. Combined LDIR and antidepressant therapy produced further improvement. Similarly, peripheral square activity differed significantly among groups (one-way ANOVA, F (4,25) = 18.82). Stressed diabetic animals showed altered peripheral activity, which was significantly improved following LDIR treatment and combined therapy, indicating reduced anxiety-like behavior and improved locomotor activity (Figure 4).

### 3.4. Rotarod test

**Figure 5.**
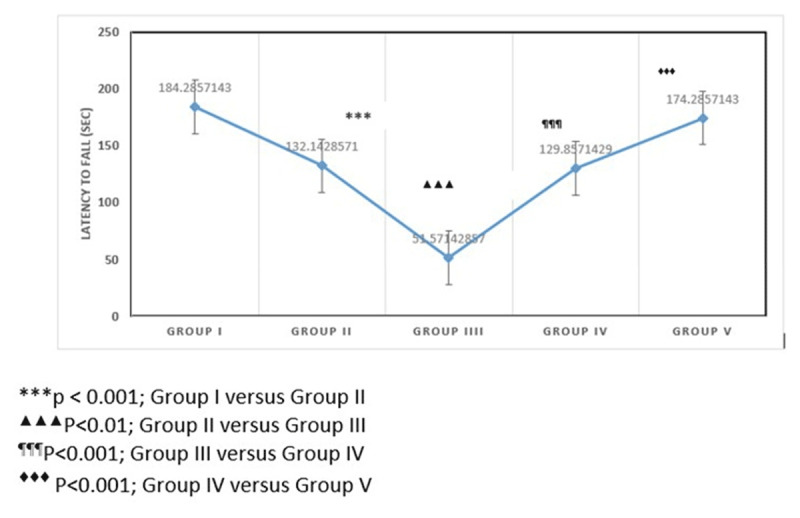
Rotarod test in the experimental groups.

Motor coordination and balance differed significantly among experimental groups (one-way ANOVA, F (4,25) = 42.32, *p* < 0.001). Diabetic rats (Group II) demonstrated impaired motor performance compared with control animals. CUS (Group III) further aggravated motor deficits, as reflected by reduced latency to fall. Treatment with low-dose ionizing radiation (Group IV) significantly improved motor performance compared with stressed diabetic animals. Furthermore, animals receiving combined LDIR and antidepressant therapy (Group V) showed additional improvement, suggesting restoration of neuromuscular coordination and motor function impaired by diabetic stress (Figure 5).

### 3.5. Object recognition test

**Figure 6.**
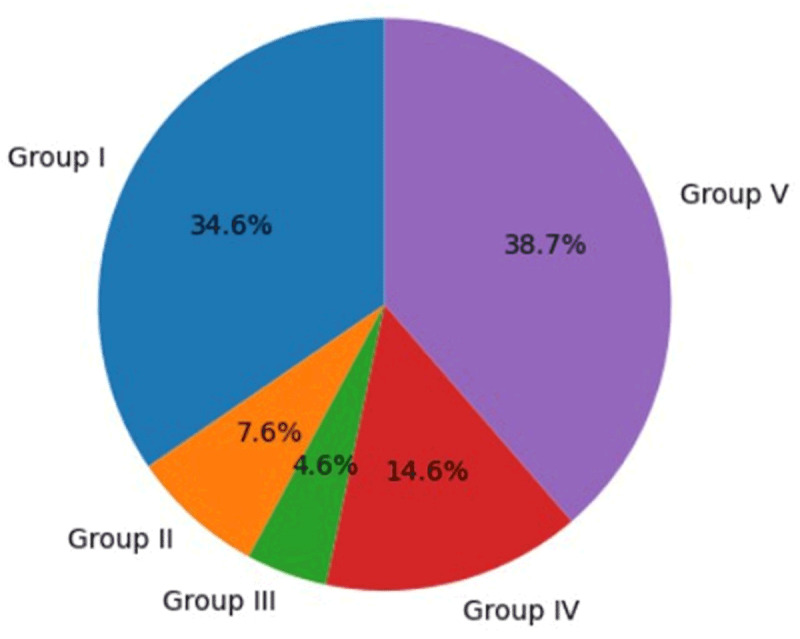
Discrimination index in the experimental groups.

Cognitive performance, assessed using the object recognition discrimination index, differed significantly among experimental groups (one-way ANOVA, F (4,25) = 111.49, *p* < 0.001). Diabetic rats (Group II) showed significant impairment in recognition memory compared with control animals. CUS (Group III) further exacerbated cognitive deficits, as reflected by reduced discrimination index values. Treatment with low-dose ionizing radiation (Group IV) significantly improved cognitive performance compared with stressed diabetic animals. Furthermore, animals receiving combined LDIR and antidepressant therapy (Group V) demonstrated additional improvement in recognition memory, suggesting enhanced cognitive recovery (Figure 6).

### 3.6. Neurotransmitter level

Dopamine levels did not differ significantly among experimental groups (one-way ANOVA, F(4,25) = 0.085), indicating that dopamine levels were not altered under the present experimental conditions (Table 1). Serotonin levels differed significantly among experimental groups (one-way ANOVA, F (4,25) = 521.37, *p* < 0.001). Chronic stress reduced serotonin levels, while LDIR treatment significantly improved serotonin concentrations (Table 1).

**Table 1.** Effect of low-dose ionizing radiation on neurotransmitter levels in hippocampus, cerebral cortex, and cerebellum.

Neurotransmitters	Group I	Group II	Group III	Group IV	Group V
HIPPOCAMPUS					
Dopamine	6.8 ± 0.55	8.5 ± 0.19	4.5 ± 0.96^▲▲▲^	27.7 ± 0.92^¶¶¶^	25.2 ± 1.36
Noradrenaline	90.9 ± 11.43	89.4 ± 4.59	78.3 ± 9.14^▲^	81.8 ± 31.49	67.0 ± 13.39
5-HT	14.8 ± 7.06	6.8 ± 0.88^*^	3.0 ± 1.51^▲▲▲^	21.7 ± 0.99^¶¶¶^	18.9 ± 0.03
Acetylcholine	18.2 ± 0.81	18.5 ± 0.91	21.1 ± 0.81	18.7 ± 1.88	18.9 ± 0.41
CEREBRAL CORTEX					
Dopamine	12.75 ± 10.62	2.69 ± 0.183^***^	2.07 ± 0.22^▲▲▲^	5.755 ± 3.88^¶^	3.62 ± 1.60
Noradrenaline	93.28 ± 18.66	83.23 ± 1.22^*^	75.80 ± 1.15^▲▲^	79.58 ± 0.030^¶^	68.6 ± 5.59
5-HT	17.08 ± 4.38	3.67 ± 7.23^***^	3.52 ± 1.20	13.58 ± 7.92^¶^	13.80 ± 9.24
Acetylcholine	18.50 ± 0.37	19.66 ± 0.12^***^	19.53 ± 0.534	21.49 ± 1.31^¶^	19.17 ± 0.55^♦^
CEREBELLUM					
Dopamine	4.10 ± 1.04	3.92 ± 0.25	1.85 ± 0.09^▲▲▲^	19.03 ± 12.18^¶¶¶^	18.79 ± 11.77
Noradrenaline	40.04 ± 12.48	87.93 ± 9.46^***^	52.29 ± 13.28^▲▲▲^	69.09 ± 6.94^¶^	55.77 ± 29.17
5-HT	18.82 ± 0.40	5.56 ± 2.82^***^	19.47 ± 0.51^▲▲▲^	20.30 ± 0.29^¶^	19.57 ± 8.76
Acetylcholine	37.38 ± 27.1	19.45 ± 0.63^***^	19.87 ± 0.83	20.99 ± 0.90	18.99 ± 0.21^♦^

^*^p < 0.05, ^***^p < 0.001; Group I vs. Group II ^▲▲^*p* < 0.05, ^▲▲^*p* < 0.01, ^▲▲▲^*p* < 0.001; Group II vs. Group III ^¶^*p* < 0.05, ^¶¶¶^*p* < 0.001; Group III vs. Group IV ^♦^*p* < 0.05; Group IV vs. Group V

Noradrenaline levels differed significantly among the experimental groups (one-way ANOVA, F(4,25) = 20.30, *p* < 0.001). LDIR treatment was associated with higher noradrenaline levels than in stressed diabetic animals (Table 1). Acetylcholine levels differed significantly among experimental groups (one-way ANOVA, F (4,25) = 6.58, *p* = 0.009), indicating significant treatment-associated neurochemical alterations (Table 1).

## 4. Discussion

The present study demonstrates that low-dose ionizing radiation (LDIR) significantly ameliorated behavioral, cognitive, and neurofunctional impairments induced by STZ-induced diabetes combined with chronic unpredictable stress (CUS). Specifically, LDIR improved spatial memory, reduced anxiety-like behavior, and enhanced motor coordination compared with untreated diabetic-stress animals. Furthermore, adjunctive paroxetine with LDIR produced greater improvements than LDIR alone, suggesting a potential synergistic or adjuvant therapeutic effect. These findings support the hypothesis that LDIR exerts neuroprotective and antidepressant-like effects, possibly through modulation of neurobiological pathways disrupted in diabetic depression.

The present study was conducted to test the hypothesis that LDIR exerts neuroprotective effects in diabetic-induced stress rats. Diabetes associated with depression involves profound pathological changes, and individuals with diabetes are more prone to developing depressive disorders. Importantly, depression in diabetic patients is often neglected, despite evidence indicating that it may act as a significant risk factor that worsens glycemic control and accelerates disease progression. Various experimental animal models have been employed to investigate behavioral, neurological, and metabolic alterations associated with depression, many of which closely resemble psychiatric manifestations observed in humans [17]. Among these, the CUS paradigm is considered a prototypical model for inducing depressive-like phenotypes.

Diabetic animal models further facilitate understanding of the complex mechanisms underlying diabetic depression. However, comparatively limited research has focused specifically on diabetic depression, and effective therapeutic strategies that prevent diabetic complications without adverse effects remain unavailable. Therefore, there is a pressing need to identify safe and potent interventions that target specific brain regions and functions involved in diabetes-associated depression.

In the present study, the antidepressant and neuroprotective potential of LDIR was explored. To date, no studies have comprehensively examined the antidepressant effects of LDIR in diabetic conditions with a particular focus on cognition, memory, anxiety-related behavior, and region-specific neurotransmitter alterations. The radiation dose used in this study falls within the established low-dose range known to induce adaptive neuroprotective responses without causing neurotoxicity, supporting its potential as a safe experimental intervention. Elevated blood glucose levels impair insulin secretion, leading to insulin resistance and increased free fatty acid mobilization via enhanced lipolysis in adipose tissue. STZ, a glucosamine nitrosourea compound, is widely used to induce diabetes in experimental animals due to its selective uptake and cytotoxicity in pancreatic β-cells [18]. The STZ dose used in this study was selected to induce stable hyperglycemia while minimizing excessive toxicity and mortality, although different dosing strategies may produce varying metabolic and neurological outcomes. Exposure to stress in diabetic rats further aggravates hyperglycemia, suggesting that stress acts as an additional risk factor contributing to glycemic instability [19]. Transient stress-induced disturbances in carbohydrate metabolism may also predispose non-diabetic individuals to the development of permanent diabetes, reinforcing the bi-directional relationship between stress and metabolic dysfunction [20]. The findings of the present study are consistent with these earlier reports.

Stress hormones are markedly elevated during both acute and chronic stress exposure and may contribute to the development of stress-induced diabetes. Persistent activation of the HPA axis and the sympathetic nervous system is believed to underlie the associated endocrine abnormalities [21]. In the present study, chronic stress exposure significantly increased serum corticosterone levels, with a more pronounced elevation in stressed diabetic rats than in unstressed diabetic controls. Notably, LDIR treatment significantly reduced blood glucose levels in stressed diabetic animals, supporting its potential antidepressant and metabolic regulatory role in diabetes.

The open field test is widely used to assess anxiety-like behavior and exploratory activity in rodents [22]. In this study, exposure to chronic unpredictable stress resulted in a marked decline in locomotor activity and exploratory behavior. Stress-induced elevations in glucocorticoids may have contributed to these behavioral impairments. Monoaminergic neurotransmitters in the hippocampus play a critical role in cognitive processing and emotional regulation, and dysregulation of biogenic amines has been strongly linked to depression [23]. Stress exposure also alters monoaminergic signaling and compromises antioxidant defense mechanisms. The present findings align with earlier reports demonstrating stress-induced monoamine depletion and oxidative imbalance in multiple brain regions [24].

Improvement in sensorimotor coordination was observed in rats treated with low-dose radiation, suggesting restoration of neuronal functional integrity disrupted by stress-induced diabetic pathology. Previous studies indicate that stress and diabetes synergistically impair motor performance through mechanisms involving oxidative stress, neuroinflammation, mitochondrial dysfunction, and reduced neuroplasticity in motor-related brain regions. Low-dose ionizing radiation is increasingly recognized as an adaptive biological stimulus that can activate endogenous protective pathways. By attenuating oxidative and inflammatory damage, enhancing mitochondrial efficiency, and supporting synaptic and neurotrophic mechanisms, LDIR may preserve neuronal integrity and functional connectivity within motor circuits. Additionally, recovery of hippocampal and prefrontal cortical function may indirectly improve motor planning and coordination. Importantly, these benefits are unlikely to result from nonspecific behavioral stimulation but rather reflect radiation-induced cellular adaptation.

The present study demonstrated significant variations in the monoaminergic and cholinergic neurotransmitter systems in diabetic rats exposed to CUS. These changes were partially restored following LDIR treatment. These findings suggest that neurotransmitter homeostasis is vulnerable to combined metabolic and stress-related insults. These findings further justify a potential modulatory effect of LDIR on neurochemical regulation.

Long-term stress and diabetes are both known to disrupt monoaminergic neurotransmission through multiple interrelated mechanisms, which eventually lead to variations of the HPA axis. Elevated corticosterone levels observed in stressed diabetic animals might contribute to neurotransmitter imbalance by impairing neuronal function, reducing neurotransmitter synthesis, and promoting neuronal vulnerability. Excess glucocorticoid exposure has been shown to affect key brain regions such as the hippocampus and cerebral cortex, which are critically involved in emotional regulation and cognitive processing [25, 26].

In the present study, serotonin levels were significantly reduced in stressed diabetic animals, which is consistent with the established role of long-term stress in declining serotonergic activity. Serotonin plays a central role in regulating mood, cognition, and neuronal plasticity. Reduced serotonin availability may result from impaired synthesis, increased reuptake, or increased degradation under conditions of metabolic and oxidative stress. A rise in blood glucose levels associated with oxidative stress might also affect tryptophan metabolism, reducing serotonin production. The observed improvement in serotonin levels following LDIR exposure suggests that low-dose radiation may support recovery of serotonergic function, possibly by activating cellular adaptive responses that enhance antioxidant defenses and stabilize neuronal metabolism.

Noradrenaline levels also showed significant alterations in diabetic stress conditions. Noradrenergic neurons are highly prone to metabolic stress due to their increased energy demands and dependence on mitochondrial function. Chronic stress impairs noradrenaline synthesis and release by disrupting the integrity of neurons, thereby reducing the enzymatic activity involved in catecholamine production. Restoration of noradrenaline levels following LDIR treatment may reflect improved neuronal resilience and enhanced cellular adaptive responses that support neurotransmitter synthesis and release.

In contrast, dopamine levels did not differ significantly among the experimental groups. This finding suggests that dopaminergic neurotransmission may be relatively less sensitive to the specific combination of metabolic and stress-related factors used in this experimental model, or that compensatory mechanisms may help preserve dopaminergic function under these conditions. Dopamine regulation is influenced by multiple interacting pathways, and regional differences in dopaminergic neuron vulnerability may contribute to variability in observed responses.

Acetylcholine levels demonstrated significant differences among experimental groups, indicating that cholinergic neurotransmission is also affected by diabetic stress. Acetylcholine has a role in cognitive function, synaptic plasticity, and neuronal communication. Metabolic dysfunction and oxidative stress may disrupt cholinergic neuron function by damaging membrane integrity and disrupting signal transmission. The observed improvement following LDIR treatment may reflect enhanced neuronal stability and improved cellular function.

The beneficial effects of low-dose ionizing radiation on neurotransmitter levels observed in this study may be explained by adaptive cellular responses induced by low-dose radiation exposure. Low-dose radiation has been reported to activate protective signaling pathways promoting cellular repair mechanisms. These adaptive responses may help to counteract oxidative and metabolic damage, protecting neuronal function and supporting neurotransmitter homeostasis [27]. Collectively, these findings suggest that combined metabolic and stress-related challenges disrupt neurotransmitter balance through neuroendocrine, metabolic, and oxidative mechanisms, whereas low-dose ionizing radiation may support neurochemical recovery by activating adaptive protective responses. However, further studies are required to investigate the precise molecular pathways underlying these effects and to evaluate the long-term safety and translational relevance of low-dose radiation exposure.

The differential regional responses observed highlight the hippocampus as the most vulnerable structure to diabetic stress, compared with the cerebellum and cortex. These regional differences may reflect variations in metabolic demand, antioxidant capacity, and receptor distribution. Restoration of neurotransmitter balance in these regions is likely to translate into improvements in affective behavior, cognition, and motor coordination.

Collectively, these findings suggest that combined metabolic and stress-related challenges disrupt neurotransmitter balance through neuroendocrine, metabolic, and oxidative mechanisms, whereas LDIR might support neurochemical recovery by activating adaptive protective responses. However, future studies incorporating oxidative stress markers, neurotrophic factors, proteomic and transcriptomic analyses, and comprehensive behavioral assessments are warranted to further elucidate the mechanistic basis and translational potential of LDIR therapy.

The findings of this study provide preliminary experimental evidence supporting the potential neuroprotective role of low-dose ionizing radiation under diabetic stress conditions. Low-dose radiation has been explored in clinical settings for its anti-inflammatory and immunomodulatory effects in selected neurological and inflammatory disorders. The dose range used in the present study falls within the low-dose exposure range known to induce adaptive protective responses without causing tissue damage. However, significant challenges remain before clinical translation, including the need for long-term safety evaluation, optimization of dosing protocols, and careful ethical and regulatory considerations. Therefore, low-dose ionizing radiation should be considered an experimental adjunct that requires further validation before potential clinical application.

The present study included both male and female rats; however, sex-specific analyses were not performed, and estrous cycle monitoring was not conducted. Hormonal fluctuations during the estrous cycle may influence stress responsiveness, neuroendocrine activity, and neurotransmitter regulation. Therefore, sex-specific differences cannot be excluded. Future studies incorporating sex-based stratification and hormonal cycle monitoring are warranted to further elucidate potential sex-dependent neuroprotective effects of low-dose ionizing radiation. Although the present study demonstrated significant neurochemical and functional alterations in diabetic rats exposed to CUS, classical behavioral paradigms specifically designed to assess depressive-like phenotypes, such as the sucrose preference test and forced swim test, were not included. Therefore, the model reflects stress-induced neurobehavioral and neurochemical alterations rather than a fully validated depression model. Additionally, morphological assessments, such as neuronal density and synaptic integrity, were not evaluated. These limitations should be considered when interpreting the findings. Future studies incorporating validated depression-specific behavioral tests and structural analyses will be necessary to further confirm the depressive-like phenotype and underlying neurobiological mechanisms. The present study did not include a diabetic-only group treated with low-dose ionizing radiation in the absence of chronic stress. This decision was made in accordance with ethical guidelines and the principle of reduction to minimize animal use while addressing the primary research objective. Consequently, the independent effects of radiation in diabetic conditions without stress cannot be fully distinguished. Future investigations incorporating additional experimental groups will be necessary to further delineate the individual and combined effects of diabetes, stress, and radiation exposure.

## 5. Conclusions

The present study demonstrates that metabolic dysfunction and CUS significantly interrupt neuroendocrine balance, behavioral performance, and neurotransmitter homeostasis in experimental animals. Elevated corticosterone levels, impaired cognitive and motor function, and alterations in serotonergic, noradrenergic, and cholinergic neurotransmission confirm the profound impact of diabetic stress on central nervous system function. Exposure to LDIR was associated with significant improvement in neuroendocrine regulation, behavioral outcomes, and selected neurotransmitter systems, particularly serotonin, noradrenaline, and acetylcholine. These findings suggest that low-dose radiation may support adaptive neuroprotective mechanisms, potentially by modulating oxidative stress, stabilizing neuroendocrine function, and preserving neuronal functional integrity. Dopaminergic neurotransmission remained relatively stable across experimental groups, indicating differential sensitivity of neurotransmitter systems to metabolic and stress-related insults. Furthermore, the combined use of LDIR and antidepressant therapy demonstrated additional improvement in behavioral and neurochemical parameters, suggesting potential adjunctive benefits. These observations support the concept that LDIR may enhance neurobiological resilience under conditions of combined metabolic and psychological stress. However, the present findings are limited to an experimental animal model, and further investigations are required to elucidate the precise molecular mechanisms, evaluate long-term safety, and determine clinical feasibility. Future studies incorporating structural, molecular, and longitudinal assessments will be essential to validate the therapeutic potential of LDIR. In conclusion, LDIR demonstrates promising experimental neuroprotective effects under diabetic stress conditions and may represent a potential adjunctive strategy for mitigating neurochemical and functional impairments associated with diabetic depression.

## Data Availability

The data presented in this study are available from the corresponding author upon reasonable request.

## List of abbreviations

CUS, chronic unpredictable stress; LDIR, low-dose ionizing radiation; STZ, streptozotocin; gm, grams; h, hours; mg/kg, milligrams per kilogram; mM, millimolar; mg/dl, milligrams per deciliter; rpm, revolutions per minute; cm, centimeters; min, minutes; Gy, Gray.
